# Characterizing the heterogeneous course of inattention and hyperactivity-impulsivity from childhood to young adulthood

**DOI:** 10.1007/s00787-021-01764-z

**Published:** 2021-04-03

**Authors:** Melissa Vos, Nanda N. J. Rommelse, Barbara Franke, Jaap Oosterlaan, Dirk J. Heslenfeld, Pieter J. Hoekstra, Marieke Klein, Stephen V. Faraone, Jan K. Buitelaar, Catharina A. Hartman

**Affiliations:** 1 Department of Psychiatry, Interdisciplinary Center Psychopathology and Emotion Regulation (ICPE), University of Groningen, University Medical Center Groningen, Groningen, The Netherlands; 2 Department of Psychiatry, Donders Institute for Brain, Cognition and Behaviour, Radboud University Medical Center, Nijmegen, The Netherlands; 3 Karakter Child and Adolescent Psychiatry University Center, Nijmegen, The Netherlands; 4 Department of Human Genetics, Donders Institute for Brain, Cognition and Behaviour, Radboud University Medical Center, Nijmegen, The Netherlands; 5 Section of Clinical Neuropsychology, Vrije Universiteit Amsterdam, Amsterdam, The Netherlands; 6 Department of Pediatrics, Emma Children’s Hospital, Amsterdam Medical Center and Vrije Universiteit Medical Center, Amsterdam, the Netherlands; 7 Department of Child and Adolescent Psychiatry, University of Groningen, University Medical Center Groningen, Groningen, The Netherlands; 8 Department of Psychiatry, UMC Utrecht Brain Center, University Medical Center Utrecht, Utrecht, The Netherlands; 9 Department of Psychiatry, State University of New York Upstate Medical University, Syracuse, NY USA; 10 Department of Neuroscience and Physiology, State University of New York Upstate Medical University, Syracuse, NY USA; 11 Department of Biomedicine, K.G. Jebsen Center for Research on Neuropsychiatric Disorders, University of Bergen, Bergen, Norway; 12 Department of Cognitive Neuroscience, Donders Institute for Brain, Cognition and Behaviour, Radboud University Medical Center, Nijmegen, The Netherlands

**Keywords:** ADHD, Heterogeneity, Trajectories, Polygenic risk scores, Late-onset

## Abstract

To advance understanding of the heterogeneity in the course of ADHD, joint symptom trajectories of inattention and hyperactivity-impulsivity from childhood to young adulthood were modelled and associated with genetic, demographic, and clinical characteristics. Data were obtained from the NeuroIMAGE cohort which includes 485 individuals with ADHD, their 665 siblings, and 399 typically developing children. Trajectories were based on scores of the Conners Parent Rating Scale Revised and estimated over seven homogeneous age bins (from 5 to 28 years) using parallel process latent class growth analysis on data collected across 2–4 time points. Multilevel multinomial logistic regression was used to identify characteristics that differentiated between the derived classes. A seven-class solution revealed “severe combined stable” (4.8%), “severe combined decreasing” (13%), “severe inattentive stable” (4.8%), “moderate combined increasing” (7.5%), “moderate combined decreasing” (12.7%), “stable mild” (12.9%), and “stable low” (44.3%) classes. Polygenic risk for depression, ADHD diagnosis, ADHD medication use, IQ, comorbid symptom levels (foremost oppositional behaviour), and functional impairment levels differentiated classes with similar ADHD symptom levels in childhood but a diverging course thereafter. The course of ADHD is highly heterogeneous, with stable, decreasing, and increasing trajectories. Overall, severe symptom levels in childhood are associated with elevated-to-severe symptom levels in adolescence and young adulthood, despite substantial symptom reductions. Beyond symptom severity in childhood, genetic, demographic, and clinical characteristics distinguish the heterogeneous course.

**Supplementary Information:**

The online version contains supplementary material available at 10.1007/s00787-021-01764-z.

## Introduction

Attention-deficit/hyperactivity disorder (ADHD) is a neurodevelopmental psychiatric disorder which is common in both children and adults. Diagnostic criteria are based on two core symptom domains: inattention and hyperactivity-impulsivity. The presence and severity of ADHD symptoms differ considerably between children with ADHD [1, 2]. Follow-up studies showed that this heterogeneity is still present in adolescence [2–5]. Most longitudinal studies so far mainly focussed on the development of symptoms in childhood and early adolescence [6–8]. This leaves important gaps of knowledge in our understanding of symptom change beyond these ages [9, 10]. A recent review that synthesized the separate childhood literature with the sparse, cross-sectional adult literature proposed hypothetical developmental trajectories of ADHD across the lifespan [6]. The authors stressed that clarification of the heterogeneous course of ADHD symptoms beyond childhood is critically needed to better inform patients, relatives, and clinicians regarding the individuals at risk for unfavourable outcomes in adulthood.

The longitudinal studies covering adolescence that have been conducted so far have yielded important insights. Several developmental subgroups have been identified, often including a group with a remitting course in addition to a group with a persisting and/or even deteriorating course [7–9]. Studies have also shown that individuals on a remitting course do not necessarily have syndromic remission (i.e., while symptoms are less severe the clinical diagnosis still applies) [7, 9, 11]. Similarly, when syndromic remission occurs individuals often continue to experience some symptoms and functional impairment [6, 12, 13]. These findings come from relatively rare longitudinal studies that used dimensional scales to examine the changes in the presence of inattention and hyperactivity-impulsivity symptoms over time [6, 7, 9, 13]. Findings that the course of ADHD can deteriorate are relevant for recent discussions whether or not adult ADHD always is a continuation of childhood ADHD [14–16]. It has been proposed that individuals with a late-onset of ADHD likely had elevated or subthreshold symptoms in childhood and developed the full syndrome in adolescence or adulthood, but this warrants further empirical research [14, 17]. A persistent, stable, course of ADHD has been associated with a higher genetic predisposition, a higher ADHD severity, and the presence of comorbid conditions [3, 4, 6, 18]. Some studies suggest a gradual relationship between such risk factors and an unfavourable course beyond childhood [10, 18]. However, as most studies only considered the persistence vs. the remittance of ADHD, the evidence for this suggested ‘dose–response’ relationship is still very scarce and can also not fully explain qualitative changes in the ADHD course such as sudden and late symptom deterioration [4, 5]. Taken together, we conclude that knowledge on the course of ADHD beyond childhood is still fragmented.

The first aim of the current study was to describe the heterogeneous course of ADHD. Data were obtained from the longitudinal NeuroIMAGE study which includes 485 individuals with ADHD, their 665 siblings with an increased vulnerability to ADHD, and 399 typically developing controls. The course of ADHD was based on scores of the cognitive problems/inattention and hyperactivity scales from the Conners Parent Rating Scale Revised and estimated over seven homogeneous age bins (between 5 and 28 years) using parallel process latent class growth analysis on data collected across 2–4 time points. Using homogeneous age bins in a heterogeneous age cohort is novel and makes our study particularly suited to disentangle momentaneous heterogeneity from developmental heterogeneity [19]. The advantage of parallel process analysis is that latent classes that diverge in the course of inattention and hyperactivity-impulsivity symptoms over time can be identified. This approach contrasts with previous studies that modelled inattention and hyperactivity-impulsivity separately [7, 9]. The second aim of this study was to identify potential differentiating characteristics of the ADHD symptom course beyond middle childhood. Multilevel multinomial logistic regression was used to examine genetic (polygenic risk for ADHD and comorbid conditions), demographic (e.g., gender and educational attainment), and clinical (comorbid symptoms and functional impairment) differences between derived classes with similar ADHD symptom levels in childhood but a diverging course thereafter.

## Methods

### Participants

The data of the multi-wave NeuroIMAGE cohort originate from the Dutch part of the International Multicentre ADHD Genetics (IMAGE) study run by the Radboud University Medical Centre Nijmegen, Vrije Universiteit Amsterdam, and University Medical Centre Groningen [20, 21]. The longitudinal study currently consists of four waves collected in 2004, 2008, 2010, and 2014 [22]. Participants were initially recruited from clinics and via advertisements. Participants were eligible if they were aged between 5 and 17 years, of European Caucasian decent, had an IQ ≥ 70, had a combined type ADHD diagnosis, had no diagnosis of autism or epilepsy, had no general learning difficulties, brain disorders or genetic disorders (such as Fragile X syndrome or Down syndrome), and had at least one biological sibling. Including newly recruited families at wave three, the complete NeuroIMAGE cohort across all four waves consists of 1549 individuals. During all waves, the participants’ behaviour off medication was assessed. All study waves were approved by the responsible ethics committees, all participants signed an informed consent, and all families received a minor financial compensation for their participation [20–22]. The NeuroIMAGE cohort is not representative of the general population. Instead, cases on the more severe end of the ADHD distribution as seen in the general population were purposely oversampled. A consequence of targeting probands with ADHD and their siblings is that, compared to the general population, the frequency of and/or number of persons within moderate and severe classes will be larger while the frequency of and/or number of persons within mild classes will be underestimated. Similar to the general population there will be a group with little to no ADHD symptoms between ages nine and twenty, as NeuroIMAGE included such a comparison sample as well. More information about the participants of the NeuroIMAGE cohort can be found in Online Resource 1.

### Measurements

#### Symptoms of ADHD and comorbid disorders

To assess symptom severity, the Conners Parent Rating Scale Revised long (CPRS-RL) was used at wave one, three, and four [23]. The CPRS-RL collects symptom scores for multiple subdomains: oppositional behaviour (10 items), cognitive problems/inattention (12 items), hyperactivity (9 items), anxious behaviour (8 items), perfectionism (7 items), social problems (5 items), psychosomatic problems (6 items), restless-impulsive behaviour (7 items), and emotional instability (3 items). To aid the interpretation of our tables and graphs, scores were rescaled to range from 0 “no symptoms” to 30 “very high symptom severity” for all subdomains. For wave two the short version of the Conners including the subdomains oppositional behaviour, cognitive problems/inattention, hyperactivity, and anxious behaviour was used. Given fewer items, and to be able to study change over time, the wave two scores were rescaled such that the range of possible scores was comparable to the range during wave one, three, and four. To ensure consistency across age bins and avoid potential rater biases, the CPRS was also used for participants above 18 years old. As a result, a total of 1513 participants filled in the CPRS at least once. Of these participants, 140 filled in the CPRS at all four waves, 645 at three waves, 305 at two waves, and 423 at only one wave. The CPRS has shown good psychometric properties [23].

#### Clinical diagnosis

At wave one, participants were assessed for ADHD by the Parental Account of Children's Symptoms (PACS) and at wave three and four by the present and lifetime version of the Kiddie Schedule for Affective Disorders and Schizophrenia (K-SADS) [20, 24]. The PACS and K-SADS are semi-structured, standardized, investigator-based interviews that are suitable to assess the presence or absence as well as the type (i.e., primarily inattentive, primarily hyperactive-impulsive or combined) of a DSM-IV ADHD diagnosis. The PACS and the K-SADS have shown good psychometric properties [20, 24].

#### Functional impairment

Functional impairment in daily life was assessed at wave one, three, and four by the impact supplement of the Strengths and Difficulties Questionnaire (SDQ) [25]. A parent-report version of the SDQ was filled in for children at all ages. Starting at age 12, participants also filled in a self-report version. The impact scale contains five items resulting in a score ranging from 0 “no functional impairment” to 10 “heavily impaired”. The impact scale of the SDQ has been validated for the assessment of longitudinal functional impairment and the SDQ has shown good psychometric properties [25, 26].

#### Polygenic risk scores (PRS)

PRS-analyses were performed using PRSice2-software [27]. GWAS summary statistics data for ADHD (20,183 cases and 35,191 controls), aggression (*N* = 18,988), antisocial behaviour (370 cases and 5850 controls), and major depressive disorder (135,458 cases and 344,901 controls) were available online (https://www.med.unc.edu/pgc/results-and-downloads, http://www.tweelingenregister.org/EAGLE/, http://broadabc.ctglab.nl/summary_statistics) [28–31]. SNPs were clumped based on linkage disequilibrium (LD) using PRSice default settings (i.e., a bi-directional 250 kb-window and R2-threshold of 0.1). PRS were generated as the standardized mean number of risk alleles in approximate linkage equilibrium, weighted by genome-wide association study allele effect size, and derived from dosage data of imputed autosomal SNPs using standard procedures. Risk alleles were defined as those associated with increased risk for ADHD, aggression, antisocial behaviour, and major depressive disorder, at a threshold of *P* < 1. This threshold was chosen to maximally capture phenotypic variance. Polygenic risk for aggression, major depressive disorder, and antisocial behaviour were chosen in line with the comorbid problems studied based on clinical relevance, i.e., externalizing, internalizing, and social problems.

Additional information on variables is provided in Online Resource 2.

### Data analysis

To ensure that homogenous developmental groups were studied the data were divided in seven age bins based on the participants’ age for each observation (≤ 9; 10–11; 12–13; 14–15; 16–17; 18–19; ≥ 20 years). This creates an optimal form of a cross-sequential design (optimal due the strong overlap of persons across age bins), see Online Resource 3.

First, developmental subgroups were identified by means of parallel processes latent class growth analysis, which is a multivariate extension of latent class growth analysis. A latent class growth analysis estimates an unique intercept and slope for a given number of latent classes, with the within class variance and covariance of the intercept and slope restricted to zero [32]. In the multivariate analysis, intercepts and slopes of inattention and hyperactivity-impulsivity are simultaneously estimated when identifying subgroups. The advantage of this simultaneous modelling is that latent classes that diverge in the course of inattention and hyperactivity-impulsivity symptoms over time can be identified. We wanted to be able to accurately estimate the points of possible symptom change. Therefore, a linear model was deemed too conservative and we permitted non-linearity in our analyses by making use of freeloading growth curves. Participants were included in the analyses if they had at least two observations (*N* = 1064; see Online Resource 1), and full information maximum likelihood was used as the estimation method. To avoid ending up with estimates at local maxima, models were run with numerous sets of initial stage random starting values (1000) and final stage optimizations (500). For each developmental subgroup, the percentage of individuals with a clinical ADHD diagnosis, comorbid symptom levels, and functional impairment levels were subsequently plotted per age bin to illustrate developmental trends.

Second, differences between the developmental subgroups in age, gender, presence of a clinical ADHD diagnosis, medication use, intelligence quotient (IQ), educational attainment, socio-economic status (SES), comorbid symptoms, functional impairment, and polygenic risk for ADHD, aggression, antisocial behaviour, and depression were examined. To make sure that potential group differences on these variables not merely reflected symptom severity differences already present in childhood, we defined our contrasts of interest as the differences between classes with a similar severity in childhood but a diverging course during adolescence and young adulthood. Class membership was predicted by each aforementioned variable separately using the three-step approach and multilevel multinomial logistic regression [33]. Variables that were assessed multiple times were averaged for these analyses. We used grand mean centring, adjusted each analysis for age, and adjusted the polygenic risk score analyses for the top ten ancestry informative genetic principal components. For all variables, except for the polygenic risk scores, missing values were handled by generating 50 imputed datasets while we reported on the pooled estimates. The maximum proportion of missing data was 26.8% for medication use, the average proportion of missing data was 5.5%. We used a significance threshold of 0.05 and did not correct for multiple testing because we considered this an explorative study with a relatively novel approach, and we did not want to miss anything with potential clinical relevance. All analyses were performed using Mplus 8 [34].

## Results

### Multivariate dimensional symptom trajectories

Based on both fit statistics and inspection of the clinical relevance of trajectories, model estimation yielded a seven-class solution that optimally described the data. Further information on the model fit is provided in Online Resource 4.

**Fig. 1:**
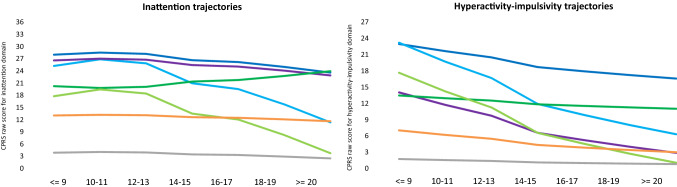
Dimensional symptom trajectories of parent-rated inattention and hyperactivity-impulsivity scores in participants of the longitudinal NeuroIMAGE study. Maximum possible score for CPRS inattention domain = 36; Maximum possible score for CPRS hyperactivity-impulsivity domain = 27. Dark blue = severe combined stable class (*N* = 48) with high levels and rather stable trajectories for both symptom domains. Light blue = severe combined decreasing class (*N* = 145) with high levels and decreasing trajectories for both symptom domains. Purple = severe inattentive stable class (*N* = 47) with a high level and stable trajectory for the inattention domain and a moderate level and decreasing trajectory for the hyperactivity-impulsivity domain. Dark green = moderate combined increasing class (*N* = 77) with a moderate level and increasing trajectory for the inattention domain and a moderate level and stable trajectory for the hyperactivity-impulsivity domain. Light green = moderate combined decreasing class (*N* = 128) with moderate levels and decreasing trajectories for both symptom domains. Orange = stable mild class (*N* = 137) with mild levels and rather stable trajectories for both symptom domains. Grey = stable low class (*N* = 482) with low levels and stable trajectories for both symptom domains

The inattention and hyperactivity-impulsivity symptom trajectories for all classes are displayed in Fig. 1. They were labelled as a “severe combined stable” class (dark blue, 4.8%) with high levels and rather stable trajectories for both domains, a “severe combined decreasing” class (light blue, 13%) with initially high levels (equal to the severe combined stable class) yet with decreasing trajectories for both domains, a “severe inattentive stable” class (purple, 4.8%) with a high level and stable trajectory for the inattention domain but moderate level and decreasing trajectory for the hyperactivity-impulsivity domain, a “moderate combined increasing” class (dark green, 7.5%) with initially moderate levels of both symptoms domains but an increasing trajectory for the inattention domain and a stable (rather than a normative decreasing) level for the hyperactivity-impulsivity domain, a “moderate combined decreasing” class (light green, 12.7%) with initially moderate levels (equal to the moderate combined decreasing class) yet decreasing trajectories for both domains, a “stable mild” class (orange, 12.9%) with mild levels and rather stable trajectories for both domains, and a “stable low” class (grey, 44.3%) with low levels and stable trajectories for both domains.

The clinical ADHD diagnosis rates approximately followed the symptom course of the different classes. This also held for comorbid externalizing and emotion dysregulation problems. Comorbid internalizing symptom levels were generally low. While most participants reported substantial functional impairment both comorbid symptoms and functional impairment decreased over time. For more information see Online Resources 5–7.

### Factors associated with the course of ADHD symptoms

**Fig. 2:**
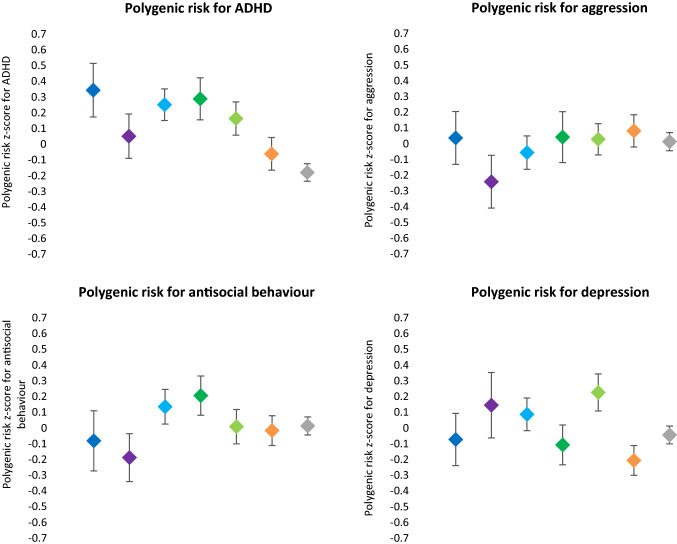
Polygenic risk scores (mean ± SE) for the seven identified dimensional symptom trajectories of parent-rated inattention and hyperactivity-impulsivity scores in participants of the longitudinal NeuroIMAGE study. Dark blue = severe combined stable class (*N* = 48) with high levels and rather stable trajectories for both symptom domains. Light blue = severe combined decreasing class (*N* = 145) with high levels and decreasing trajectories for both symptom domains. Purple = severe inattentive stable class (*N* = 47) with a high level and stable trajectory for the inattention domain and a moderate level and decreasing trajectory for the hyperactivity-impulsivity domain. Dark green = moderate combined increasing class (*N* = 77) with a moderate level and increasing trajectory for the inattention domain and a moderate level and stable trajectory for the hyperactivity-impulsivity domain. Light green = moderate combined decreasing class (*N* = 128) with moderate levels and decreasing trajectories for both symptom domains. Orange = stable mild class (*N* = 137) with mild levels and rather stable trajectories for both symptom domains. Grey = stable low class (*N* = 482) with low levels and stable trajectories for both symptom domains

Means and standard deviations of the demographic and clinical factors are listed per class in Table 1. The polygenic risk scores are listed in Fig. 2, with *z* scores given per class. Visual inspection of Table 1, Fig. 2 and Online Resource 8 showed that the severity of most genetic, demographic, and clinical variables gradually increased alongside the relative severity of the identified classes. Polygenic risk for ADHD, polygenic risk for aggression, polygenic risk for antisocial behaviour, polygenic risk for depression, IQ, and SES did not significantly differ for our contrasts of interest, but did deviate from this general pattern.

**Table 1.** Demographic and clinical descriptives for the seven identified dimensional symptom trajectories of parent-rated inattention and hyperactivity-impulsivity scores in participants of the longitudinal NeuroIMAGE study.

	1		2		3		4		5		6		7	
	Severe combined stable		Severe inattentive stable		Severe combined decreasing		Moderate combined increasing		Moderate combined decreasing		Stable mild		Stable low	
	*N* = 48		*N* = 47		*N* = 145		*N* = 77		*N* = 128		*N* = 137		*N* = 482	
	Mean	SD	Mean	SD	Mean	SD	Mean	SD	Mean	SD	Mean	SD	Mean	SD
Descriptives														
Age	16.4	2.6	14.1	2.1	15.6	3.0	14.6	2.9	13.8	2.6	14.1	3.2	14.8	3.5
Gender, % males (*N*)	89.6	43	80.9	38	79.3	115	74.0	57	68.8	88	55.5	76	37.8	182
ADHD diagnosis, % lifetime diagnosis (*N*)	100.0	48	100.0	47	98.6	143	97.4	75	91.4	117	63.5	87	5.0	24
ADHD medication, % lifetime use (*N*)	87.5	42	76.6	36	75.2	109	72.2	56	67.2	86	32.1	44	0.4	2
Status, % controls (*N*)	0.0	0	0.0	0	0.0	0	1.3	1	1.6	2	12.4	17	49.2	237
Status, % siblings (*N*)	12.5	6	40.4	19	18.6	27	41.6	32	29.7	38	64.2	88	50.8	245
Status, % probands (*N*)	87.5	42	59.6	28	81.4	118	57.1	44	68.8	88	23.4	32	0.0	0
IQ	93.2	13.8	100.9	12.7	97.5	13.2	98.3	12.5	97.0	13.8	100.3	13.9	104.7	11.4
SES	11.3	2.1	12.2	2.5	11.5	2.4	10.9	2.1	11.4	2.4	11.6	2.3	12.1	2.6
Comorbid symptoms														
Oppositional behaviour	16.5	4.1	9.3	4.6	11.9	4.7	11.7	4.1	9.4	4.2	6.6	3.6	2.8	2.5
Emotional instability	16.0	5.4	8.9	7.1	10.4	6.9	9.5	5.3	8.8	6.1	5.5	5.0	2.1	3.2
Anxious behaviour	7.7	5.9	6.1	5.4	6.1	5.4	4.9	4.4	5.2	5.0	4.3	4.6	2.4	2.8
Perfectionism	8.0	4.7	4.1	3.6	6.3	5.1	4.4	4.4	5.0	4.0	3.8	3.6	3.2	3.1
Social problems	10.8	6.6	8.2	6.3	8.9	6.8	6.6	5.3	5.8	6.0	4.7	5.1	1.5	2.2
Functional impairment														
Educational attainment	2.1	0.6	2.3	0.8	2.2	0.8	2.2	0.7	2.3	0.8	2.3	0.7	2.8	0.9
Parent-reported functional impairment	4.8	2.2	3.2	1.7	3.3	2.0	3.1	1.8	2.4	1.7	1.5	1.5	0.2	0.6
Self-reported functional impairment	1.5	1.8	1.0	1.1	1.4	1.7	1.3	1.5	1.0	1.2	0.9	1.3	0.3	0.9

Presented are the mean and standard deviation averaged across all four waves. Contrasts of interest are 1 vs. 2, 1 vs. 3, 2 vs. 3, and 4 vs. 5. The educational attainment score ranges from 0 “no formal education” to 4 “scientific education”. Maximum possible SES = 17; maximum possible rescaled score for each CPRS subdomain = 30; maximum possible SDQ impact score = 10

*IQ* intelligence quotient, *SES* socio-economic status, *SD* standard deviation

The seven trajectories yielded four contrasts of interest with similar severity levels in childhood: (1) the severe combined stable was compared with severe inattentive stable class, (2) the severe combined stable was compared with the severe combined decreasing class, (3) the severe inattentive stable was compared with severe combined decreasing class, and (4) the moderate combined increasing class was compared with the moderate combined decreasing class. The odds ratios and p-values for the contrasts of interest for genetic, demographic, and clinical factors are provided in Table 2.

**Table 2.** Multinomial logistic regression results for the contrasts of interest among the seven identified dimensional symptom trajectories of parent-rated inattention and hyperactivity-impulsivity scores in participants of the longitudinal NeuroIMAGE study

	Severe combined stable (*N* = 48) vs. severe combined decreasing (*N* = 145)		Severe inattentive stable (*N* = 47) vs. severe combined decreasing (*N* = 145)		Severe combined stable (*N* = 48) vs. severe inattentive stable (*N* = 47)		Moderate combined increasing (*N* = 77) vs. moderate combined decreasing (*N* = 128)	
	Odds ratio (95% CI)	*p*	Odds ratio (95% CI)	*p*	Odds ratio (95% CI)	*p*	Odds ratio (95% CI)	*p*
Descriptives								
Age	1.08 (0.99–1.18)	0.10	0.85 (0.78–0.93)	< 0.0001	1.27 (1.15–1.40)	< 0.0001	1.11 (1.00–1.22)	0.04
Gender (1 = male; 2 = female)	0.42 (0.14–1.32)	0.14	0.84 (0.29–2.46)	0.75	0.50 (0.13–2.00)	0.33	0.28 (0.32–1.80)	0.53
ADHD diagnosis (0 = no diagnosis; 1 = diagnosis)	51.52 (1.60–1657.66)	0.03	4.91 (0.10–251.31)	0.43	10.50 (0.07–1548.56)	0.36	2.91 (0.40–21.15)	0.29
ADHD medication (0 = no medication; 1 = medication)	31.34 (1.28–766.44)	0.04	4.29 (0.04–440.75)	0.54	7.31 (0.04–1229.55)	0.45	2.78 (0.05–168.21)	0.63
IQ	0.97 (0.94–1.00)	0.06	1.03 (0.99–1.06)	0.13	0.95 (0.91–0.98)	0.01	1.01 (0.98–1.05)	0.41
SES	0.96 (0.82–1.13)	0.65	1.14 (0.96–1.34)	0.13	0.85 (0.70–1.03)	0.09	0.92 (0.78–1.10)	0.35
Polygenic risk scores								
ADHD	1.08 (0.71–1.65)	0.72	0.69 (0.43–1.10)	0.12	1.56 (0.95–2.58)	0.08	1.27 (0.74–2.19)	0.38
Aggressive behaviour	1.20 (0.76–1.89)	0.44	0.69 (0.39–1.21)	0.19	1.74 (0.98–3.07)	0.06	1.21 (0.66–2.21)	0.55
Antisocial behaviour	0.79 (0.47–1.35)	0.39	0.69 (0.42–1.14)	0.15	1.14 (0.64–2.02)	0.65	1.52 (0.83–2.81)	0.18
Depression	0.84 (0.54–1.30)	0.43	1.21 (0.48–3.04)	0.69	0.69 (0.26–1.88)	0.47	0.48 (0.27–0.85)	0.01
Comorbid symptoms								
Oppositional behaviour	1.34 (1.21–1.49)	< 0.0001	0.82 (0.72–0.92)	0.00	1.64 (1.41–1.91)	< 0.0001	1.19 (1.08–1.32)	0.00
Emotional instability	1.79 (1.43–2.24)	< 0.0001	0.79 (0.60–1.05)	0.11	2.25 (1.64–3.10)	< 0.0001	1.11 (0.93–1.33)	0.25
Anxious behaviour	1.10 (1.02–1.19)	0.02	0.98 (0.89–1.07)	0.60	1.13 (0.80–0.98)	0.02	0.99 (0.90–1.10)	0.90
Perfectionism	1.11 (1.01–1.23)	0.04	0.82 (0.71–0.95)	0.01	1.35 (1.16–1.57)	< 0.0001	0.91 (0.77–1.08)	0.29
Social problems	1.12 (1.01–1.23)	0.03	0.93 (0.83–1.05)	0.27	1.20 (1.04–1.37)	0.01	1.10 (0.94–1.28)	0.23
Functional impairment								
Educational attainment	0.87 (0.46–1.62)	0.65	1.28 (0.71–2.33)	0.41	0.67 (0.33–1.39)	0.28	0.81 (0.49–1.35)	0.42
Parent-reported functional impairment	1.54 (1.30–1.95)	< 0.0001	0.91 (0.73–1.13)	0.41	1.68 (1.33–2.29)	< 0.0001	1.24 (0.99–1.62)	0.07
Self-reported functional impairment	1.16 (0.88–1.37)	0.40	0.83 (0.60–1.03)	0.08	1.39 (1.03–1.88)	0.03	1.11 (0.84–1.47)	0.45

Significance level = 0.05. The educational attainment score ranges from 0 “no formal education” to 4 “scientific education”. Maximum possible SES = 17; maximum possible rescaled score for each CPRS subdomain = 30; maximum possible SDQ impact score = 10

*IQ* intelligence quotient, *SES* socio-economic status, *CI* confidence interval

The severe combined stable class differed from the severe inattentive stable class by a lower IQ, higher comorbid oppositional behaviour, emotional instability (i.e., mood changes and temper outbursts), anxious behaviour, perfectionism, and social problem symptom levels.

The severe combined stable and severe inattentive stable classes had both higher clinical ADHD rates, lifetime medication use, and functional impairment levels compared to the severe combined decreasing class. The severe combined stable class additionally differed from the severe combined decreasing class by higher comorbid oppositional behaviour, emotional instability, anxious behaviour, perfectionism, and social problem symptom levels. The severe inattentive stable class additionally differed from the severe combined decreasing class by higher comorbid oppositional behaviour symptom levels.

The moderate combined increasing class differed from the moderate combined decreasing class by a lower polygenic risk for depression and higher comorbid oppositional behaviour symptom levels.

The remaining variables (i.e., polygenic risk for ADHD, aggression, antisocial behaviour, and gender, SES, and educational attainment) did not significantly differ between the classes with similar childhood ADHD symptom levels.

## Discussion

The current study examined (1) the heterogeneity in the joint course of inattention and hyperactivity-impulsivity from childhood to young adulthood and (2) the factors that differentiated trajectories with similar symptom severity in childhood but a diverging course thereafter. The study delivered several key findings. With respect to aim (1) we showed that the course of inattention and hyperactivity-impulsivity during adolescence was very heterogeneous. Among the identified trajectories were groups of individuals with stable high, decreasing, and increasing symptom levels over time. In our sample, 9.6% had persistently high levels of both inattention and hyperactivity-impulsivity, or attention problems only. For around 26% symptom levels decreased, although the clinical ADHD diagnosis still applied in most cases (52.2%). Among those with an increasing symptom course, 18.6% had an adolescent-onset of clinical ADHD. This late-onset resulted from a gradually progressing ADHD symptom severity during adolescence and was unrelated to an onset of comorbid conditions. With respect to aim (2) it was found that most demographic and clinical factors, which are known to associate with the course of ADHD, correlated in a ‘dose–response’ relationship with the symptom severity of the identified trajectories. Beyond this general pattern aligned with ADHD symptom severity, polygenic risk for depression, ADHD diagnosis, ADHD medication use, IQ, comorbid symptom levels (foremost oppositional behaviour), and functional impairment levels differentiated classes with similar ADHD symptom levels in childhood but a diverging course thereafter.

Within the increasing trajectory, a fourth of the patients had an adolescent-onset of ADHD. This is in line with recent research suggesting that ADHD can manifest beyond childhood [14–16]. In accordance with earlier research, our patients with late-onset ADHD already experienced subthreshold symptoms [35, 36]. In contrast to earlier findings, the increase in ADHD symptoms was not related to the presence or emergence of comorbid symptoms in our cohort (see Online Resource 6 for more information) [17, 36]. In adolescence important changes in the environment occur while compensating factors present during childhood attenuate (e.g., school expects strong planning abilities at the same time that parents reduce their help therein). It has been proposed that this may result in symptom deterioration and adolescent clinical onsets of children with subthreshold problems who are at risk [17, 35, 37]. We conclude from our findings that an adolescent onset of clinical ADHD is not sudden, but gradual and preceded by subthreshold problems, and does not coincide with an onset of other disorders. The suggestion that adolescent onset ADHD in persons with subthreshold symptoms may surface due to reduced scaffolding by parents has to be empirically studied.

Genetic, demographic, and clinical correlates of the course of ADHD generally followed the overall symptom severity of the identified classes in a ‘dose–response’ relationship. This is consistent with existing literature focussed on the presence or absence of ADHD, showing that persons with persistent ADHD symptoms had the most comorbid problems and functional impairment [12, 14, 38]. We showed that oppositional behaviour and emotional instability symptoms (i.e., mood fluctuations and temper outbursts) were most strongly associated with the course severity of ADHD symptoms, in particular the course of hyperactivity-impulsivity. Our finding is in line with the poor self-regulation inherent to the disorder that manifests in behavioural, cognitive, and emotional symptoms that are broader than the DSM criteria of ADHD. Consistent with the literature, we conclude that symptom severity in childhood, as well as correlates of this severity like comorbid oppositional and emotion dysregulations problems, and functional impairment, are important markers of the ADHD symptom course [4, 12, 14, 38].

Genetic and environmental risk factors that did not follow the ADHD symptom course were polygenic risk for ADHD, aggression, antisocial behaviour, and depression, IQ, and SES. First, in the severe inattentive stable class the polygenic risk for ADHD was remarkably low and the polygenic risk for depression relatively high. Possibly, the ADHD symptoms of the individuals that belong to this “inattentive only” class have a different etiological background than both remitting (both hyperactivity-impulsivity and inattention become less severe over time) and persistent ADHD (both hyperactivity-impulsivity and inattention remain substantial over time). The particularly high polygenic risk for depression in persons of whom the ADHD symptoms remitted, indicates that this might be a subgroup of persons with genetic vulnerability to both ADHD and depression that are at risk of future comorbid depression, in line with the high genetic correlation between ADHD and depression [28].

We are among the very first to do this kind of detailed developmental work. While this is an asset, our study had several limitations. The current study was longitudinal, but the age at entry, age when data contribution ended, and the number of data points differed between participants. This was handled by creating an optimal form of a cross-sequential design using same age bins. While this gave the opportunity to examine homogenous developmental groups and model trajectories over a much longer developmental period, some caution is necessary when interpreting data in terms of longitudinal stability and change. This, because not all participants were followed from below nine years old to above twenty years old as is inherent to the cross-sequential design. In addition, the cross-sequential design includes probands with an older age at entry, which might have led to an overestimation of the number of persons in the severe combined stable class when compared to a longitudinal design of same aged individuals. This, since adolescents who still have inattention and/or hyperactivity-impulsivity symptoms are, by definition, not among the subgroup that remits during this age. The primary focus of this paper was to comprehensively chart and describe the heterogeneous course of ADHD. To be able to identify relatively rare symptom trajectories (e.g., the increasing trajectory) the identification of small classes (smallest class *N* = 47) was permitted. Yet, this decreased statistical power to detect differentiating factors, which was the second focus of our study. Even larger samples would be needed to further characterize the less common trajectories. Last, we cannot exclude the possibility of reporting false positive differences, as we did not correct for multiple testing, to keep statistical power high. Despite these limitations, the value of using the (few) existing longitudinal cohorts that reach adulthood to enhance understanding of the heterogeneity of ADHD beyond childhood cannot be overstated and we pursued this, in our view, in the best possible way.

## Conclusions

We conclude that the course of ADHD is highly heterogeneous. Individuals with (subthreshold levels of) ADHD symptoms show different symptom trajectories with partly disjoint changes of inattention and hyperactivity-impulsivity over time. Individuals following a progressive symptom trajectory, part of whom had an adolescent-onset ADHD diagnosis, already had subthreshold levels of ADHD. In addition to childhood symptom severity of ADHD, polygenic risk for depression, ADHD diagnosis, ADHD medication use, IQ, comorbid problems, and functional impairment differentiated classes with similar ADHD symptom levels in childhood but a diverging course thereafter. Environmental factors, such as parental scaffolding, were currently not studied but may also explain part of the ADHD course. The heterogeneity in the course of ADHD identified in this study underlines the necessity of monitoring and tailored care for individuals with subthreshold as well as clinical ADHD.

## Supplementary Information

Below is the link to the electronic supplementary material.

Supplementary file1 (PDF 485 kb)
