# Supplementary material for: Characterizing the heterogeneous course of inattention and hyperactivity-impulsivity from childhood to young adulthood
Source: Eur Child Adolesc Psychiatry. 2021 Apr 3;31(8):1–11. doi: 10.1007/s00787-021-01764-z (PMC9343304; doi:10.1007/s00787-021-01764-z)
Supplement: Supplementary file 1 — Supplementary file1 (PDF 485 kb) [file 787_2021_1764_MOESM1_ESM.pdf]

SUPPLEMENTARY MATERIAL

Online Resource 1. Additional information about the participants of the NeuroIMAGE cohort.

Including newly recruited families at wave three, the complete NeuroIMAGE cohort across all four waves consists of 1,549 individuals. Of these individuals, 213 participated in all four waves, 721 in three waves, 272 in two waves, and 343 in only one wave. For 80.9% of the families it was possible to collect data of two or more siblings. The retention rate from the original IMAGE study for at least three waves was high: 76.3%. The loss of participation for one or several waves is primarily explained by the discontinuation of one institute from wave three to wave four (72.5%). Other reasons often given were: being too busy, family problems, and the time consumption of the study.

Of the participants of the NeuroIMAGE cohort 1,064 had at least two observations for the CPRS-R and were included in our analyses. Participants that were included in the analyses did not differ from those that were excluded when looking at intelligence quotient ($M = 101.14$ vs. 100.14; $t(383) = 0.992$; $p = 0.322$, socio-economic status ($M = 11.74$ vs. 11.97; $t(1091) = -1.101$; $p = 0.271$), ADHD symptom severity ($M = 7.90$ vs. 7.39; $t(1511) = 1.395$; $p = 0.163$), or overall symptom severity ($M = 4.71$ vs. 5.14; $t(681) = -1.857$; $p = 0.064$).

Online Resource 2. Additional information on variables.

Medication. The participant's history of psychoactive treatment was assessed at wave one, three, and four using pharmacy prescription records [1]. If pharmacy prescription records were not available, treatment history was reconstructed via face-to-face semi-structured parental interviews. The current study took into account whether a participant did or did not use ADHD medication (i.e., methylphenidate and atomoxetine) up to four months prior to each assessment day [1].

Intelligence quotient. At wave one, three, and four the intelligence quotient of all participants was estimated. At wave one the vocabulary, similarities, picture completion and block design subtests from the third edition of the Wechsler Intelligence Scale for Children (WISC) or the Wechsler Adult Intelligence Scale (WAIS) (depending on the subjects' age) were used [2,3]. At wave three and four only the vocabulary and block design subtests were used. The subtests correlate between 0.90 and 0.95 with full-scale IQ [4].

Socio-economic status. The participants' socio-economic status was computed by recoding and averaging the highest successfully completed education level of both parents into a measure reflecting their combined years of formal education [5]. This scale contained nine levels for each parent and combined ranges from 0 ''no formal education'' to 17 ''university'' [5].

Educational attainment. To asses educational attainment the current educational track or highest finished level of education of all participants was retrieved. The educational attainment scores include 0 ''no formal education'', 1 ''lower education'', 2 ''applied education'', 3 ''professional education'', and 4 ''scientific education'' [6].

Genotyping. DNA isolation was performed at the Radboudumc Nijmegen, according to Manufacturer's protocols. Genotyping was performed using the Illumina Psych-Array 24 v1.1A and genotyping data was imputed using the RICOPILI-pipeline [7]. Only single nucleotide polymorphisms (SNPs) passing quality control filters regarding imputation quality > 0.8, minor allele frequency (MAF) >0.01), Hardy-Weinberg equilibrium test (P cut-off 1x10-6) and SNP-call rate (0.95) were retained. Individual genome-wide genotype data was available for 5,064,466 SNPs for 780 subjects in NeuroIMAGE.

REFERENCES

[1] Schweren LJS, Groenman AP, von Rhein D, Weeda W, Faraone S V., Luman M, et al. Stimulant Treatment Trajectories Are Associated With Neural Reward Processing in Attention-Deficit/Hyperactivity Disorder. J Clin Psychiatry 2015;77:22–7. doi:dx.doi.org/10.4088/JCP.14m09658.

[2] Wechsler D. Wechsler Intelligence Scale for Children. Third. London: The Psychological Corporation; 2002.

[3] Wechsler D. Wechsler Adult Intelligence Scale. Third. London: The Psychological Corporation; 2000.

[4] Groth-Marnat G. Handbook of psychological assessment. Third. New York: Wiley; 1984.

[5] Buis ML. Inequality of Educational Outcome and Opportunity in the Netherlands during the 20th Century. Amsterdam: VU-University; 2010.

[6] Esch P, Bocquet V, Pull C, Couffignal S, Lehnert T, Graas M, et al. The downward spiral of mental disorders and educational attainment: A systematic review on early school leaving. BMC Psychiatry 2014;14:1–13. doi:10.1186/s12888-014-0237-4.

[7] Lam M, Awasthi S, Watson HJ, Goldstein J, Panagiotaropoulou G, Trubetskoy V, et al. RICOPILI: Rapid Imputation for COnsortias PIpeLIne. Bioinformatics 2019:1–4. doi:10.1093/bioinformatics/btz633.

Online Resource 3. Methodological background of the age bin allocation.

The optimal number and width of the age bins were determined using the frequency distribution of the age of the participants, and considered relevance and covariance coverage. Bins should be small enough to prevent the allocation of two measurements to the same bin, and large enough to ensure a sufficient sample size per bin for each estimated trajectory. Because we wanted to ensure a sufficient sample size per bin, and the age within the NeuroIMAGE cohort is normally distributed, we chose to permit larger bins at the two extremes. Though we chose the width of our age bins as optimal as possible, some participants still ended up with multiple measurements in one age bin. Two different approaches were used to make the best out of this drawback. First, if participants ended up with multiple measurements in one bin due to the extended age ranges at the extremes, their CPRS scores were averaged. The scores were averaged because this enhances the reliability of a measurement. Second, if participants ended up with multiple measurements in one bin due to the short time between wave 2 and wave 3, the third CPRS-RL measurement was favoured over the second CPRS-RS measurement. The third measurement was favoured because 1) more items were used to measure the ADHD subdomains and 2) scores for additional psychopathology subdomains were present.

Online Resource 4. Fit statistics for the estimated parallel process latent class growth models.

Model fit was assessed with the Akaike Information Criterion, Bayesian Information Criterion, entropy score, Vuong Lo Mendell Rubin Test, and Bootstrapped Likelihood Ratio Test as is common practice. With a sample size as large as ours, statistical indices may in part be inconclusive, such that more refined models may keep showing a better fit, with the risk of overfitting the data. For final model selection, decisions were therefore additionally based on the clinical relevance of new trajectories as well as the percentages of participants in the smallest class. New trajectories were considered clinically relevant when characterization of the trajectory would strongly add to the current literature.

The two model fit indices that showed distinctive model fits between solutions were the entropy score and the Vuong Lo Mendell Rubin test. Both the entropy score and the Vuong Lo Mendell Rubin test initially favoured the three-class solution, consisting of two remitting ADHD classes and a typically developing class, yet the entropy increased again for the seven-class solution. The seven-class solution yielded six clinically relevant classes in addition to a healthy comparison group and the smallest class was 4.8% percent which still pertains to a substantial number of children. Combining this information with the second entropy peak for the seven-class solution, the seven-class model was considered optimal.

Table S1. Fit statistics for the estimated parallel process latent class growth models of parent-rated inattention and hyperactivity-impulsivity scores in participants of the longitudinal NeuroIMAGE study.

**Model**	***AIC***	***BIC***	***Entropy***	***VLMR***	***BLRT***	***Clinically relevant***	***Smallest class, %***
1 class	38467.94	38607.10	x	x	x	Yes	100.0
2 class	34968.54	35132.55	0.946	<0.05	<0.05	Yes	42.1
3 class	34162.73	34351.58	0.911	<0.05	<0.05	No	15.3
4 class	33888.84	34102.54	0.872	0.07	<0.05	Yes	7.6
5 class	33769.73	34008.28	0.848	0.52	<0.05	Yes	5.0
6 class	33627.66	33891.06	0.847	0.43	<0.05	Yes	6.1
7 class	33503.04	33791.29	0.859	0.16	<0.05	Yes	4.8
8 class	33430.19	33743.29	0.854	0.59	<0.05	No	4.2

Notes: AIC = Akaike Information Criterion; BIC = Bayesian Information Criterion.

VLMR = Vuong Lo Mendell Rubin likelihood ratio test; BLRT = Parametric bootstrapped likelihood ratio test.

Figure S1. Estimated versus observed dimensional symptom trajectories of parent-rated inattention and hyperactivity-impulsivity scores in participants of the longitudinal NeuroIMAGE study.

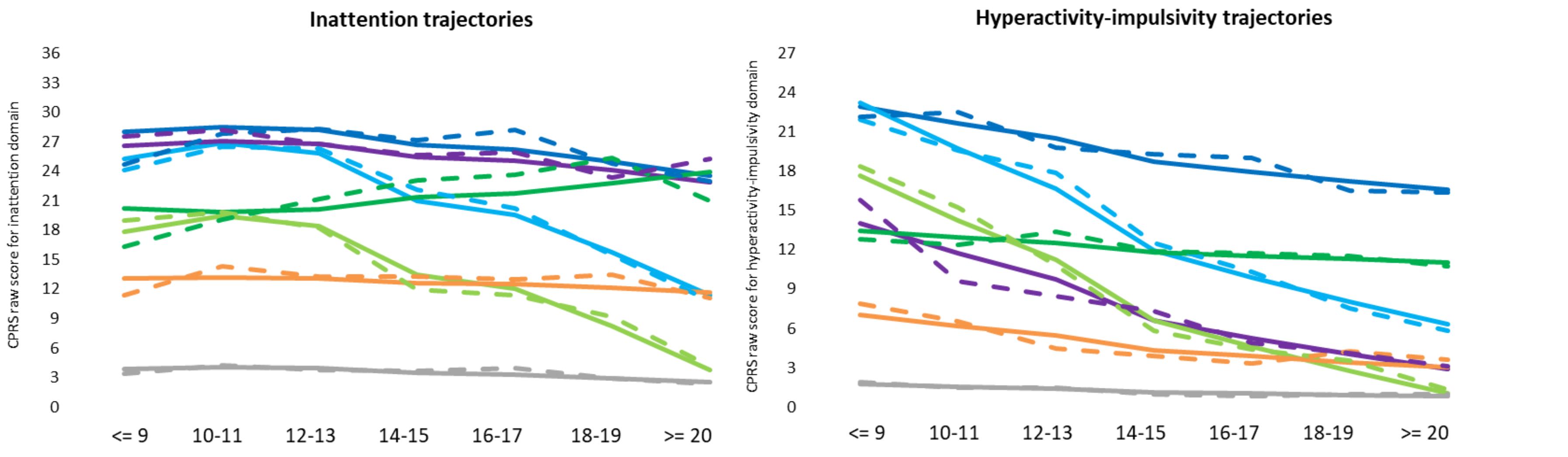

Notes: Maximum possible score for CPRS inattention domain = 36; Maximum possible score for CPRS hyperactivity-impulsivity domain = 27.

The solid lines refer to the estimated dimensional symptom trajectories, the dashed lines refer to the observed dimensional symptom trajectories.

Dark blue = Severe combined stable class (N=48) with high levels and rather stable trajectories for both symptom domains.

Light blue = Severe combined decreasing class (N=145) with high levels and decreasing trajectories for both symptom domains.

Purple = Severe inattentive stable class (N=47) with a high level and stable trajectory for the inattention domain and a moderate level and decreasing trajectory for the hyperactivity-impulsivity domain.

Dark green = Moderate combined increasing class (N=77) with a moderate level and increasing trajectory for the inattention domain and a moderate level and stable trajectory for the hyperactivity-impulsivity domain.

Light green = Moderate combined decreasing class (N=128) with moderate levels and decreasing trajectories for both symptom domains.

Orange = Stable mild class (N=137) with mild levels and rather stable trajectories for both symptom domains.

Grey = Stable low class (N=482) with low levels and stable trajectories for both symptom domains.

Online Resource 5. Clinical diagnosis rates over time.

The course of clinical ADHD diagnosis rates for all classes is plotted in Figure 2. Class differences were most apparent in young adulthood. The severe combined stable trajectory had a very high and stable rate of participants with an ADHD diagnosis. This also held for the severe inattentive stable and severe combined decreasing classes up to late adolescence after which clinical diagnosis rates decreased with respectively 23% (87 to 64%) and 24%. The moderate combined decreasing class showed a decreasing rate of participants with a clinical diagnosis of 55%. The moderate combined increasing class showed an increasing rate of participants with a clinical diagnosis of 24%; indicating part of the individuals in this class had a late clinical onset, after age 15.

Figure S2. Clinical ADHD diagnosis trajectories of investigator based clinical diagnosis rates in participants of the longitudinal NeuroIMAGE study.

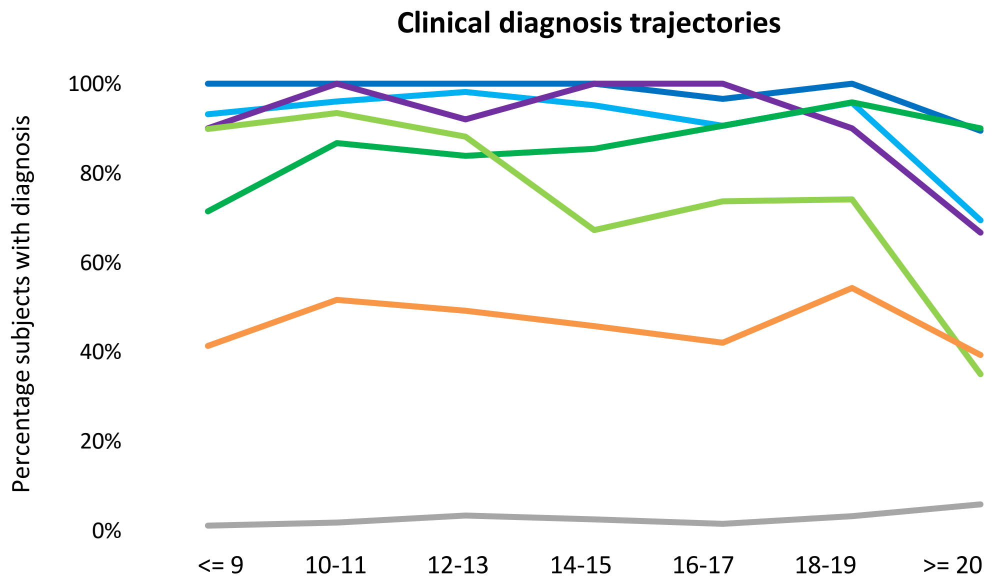

Notes: Dark blue = Severe combined stable class (N=48) with high levels and rather stable trajectories for both symptom domains.

Light blue = Severe combined decreasing class (N=145) with high levels and decreasing trajectories for both symptom domains.

Purple = Severe inattentive stable class (N=47) with a high level and stable trajectory for the inattention domain and a moderate level and decreasing trajectory for the hyperactivity-impulsivity domain.

Dark green = Moderate combined increasing class (N=77) with a moderate level and increasing trajectory for the inattention domain and a moderate level and stable trajectory for the hyperactivity-impulsivity domain.

Light green = Moderate combined decreasing class (N=128) with moderate levels and decreasing trajectories for both symptom domains.

Orange = Stable mild class (N=137) with mild levels and rather stable trajectories for both symptom domains.

Grey = Stable low class (N=482) with low levels and stable trajectories for both symptom domains.

Online Resource 6. Comorbid symptom levels over time.

The course of comorbid symptoms for all classes is plotted in Figure 3. Class differences in externalizing comorbid symptoms (oppositional behaviour and emotional instability) were apparent at all ages, with high symptom levels for the severe combined stable class already present in childhood. Oppositional behaviour levels slightly decreased in the severe combined stable and severe inattentive stable classes, robustly decreased in the two decreasing classes, and increased transiently in the moderate combined increasing class. Emotional instability levels decreased in all classes. Social problem levels decreased in most classes, except for stable trajectories in the severe inattentive stable and moderate combined increasing classes. Internalizing comorbid symptoms (anxious behaviour and perfectionism) were only mildly apparent at any age, with low perfectionism levels in the severe combined stable class in childhood with a mild peak level at early adolescence. Anxious behaviour levels slightly decreased in all classes. Class differences in social problem levels were apparent at all ages.

Figure S3. Comorbid symptom trajectories of parent-rated oppositional behaviour, emotional instability, social problems, anxious behaviour, and perfectionism scores in participants of the longitudinal NeuroIMAGE study.

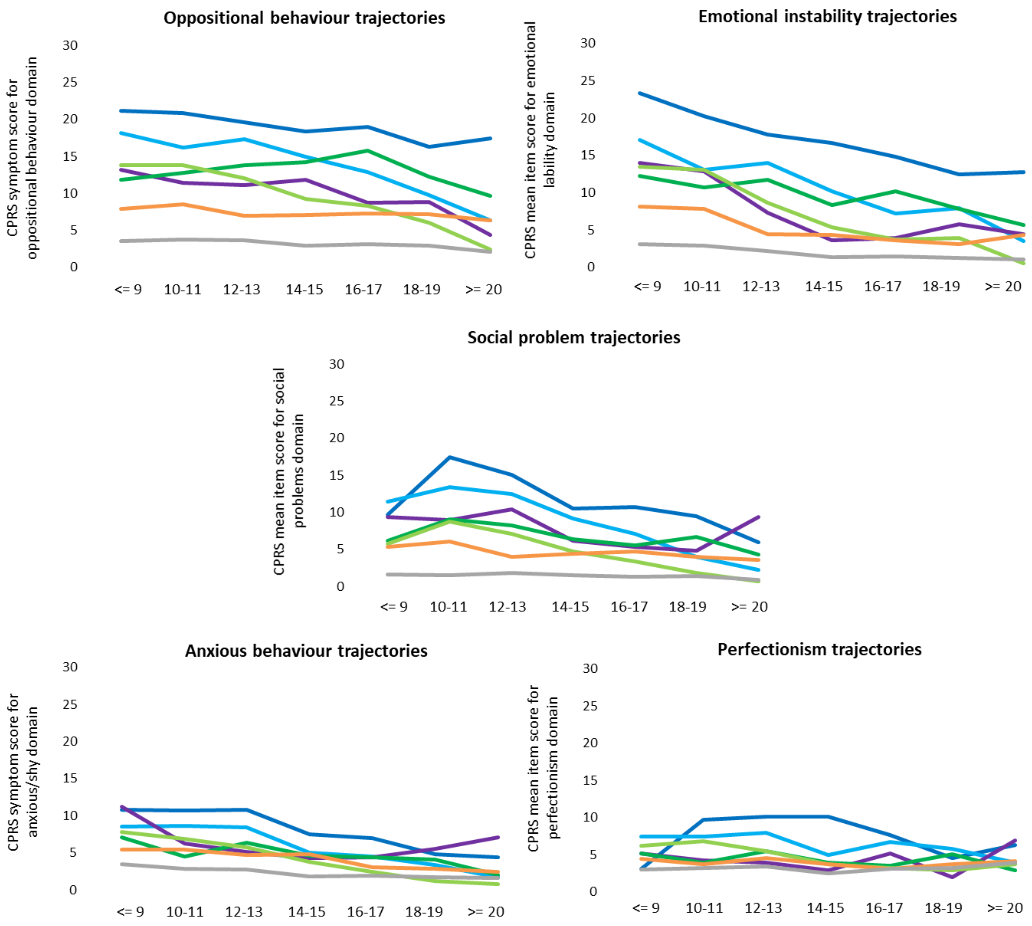

Notes: Maximum possible rescaled score for each CPRS domain = 30.

Dark blue = Severe combined stable class (N=48) with high levels and rather stable trajectories for both symptom domains.

Light blue = Severe combined decreasing class (N=145) with high levels and decreasing trajectories for both symptom domains.

Purple = Severe inattentive stable class (N=47) with a high level and stable trajectory for the inattention domain and a moderate level and decreasing trajectory for the hyperactivity-impulsivity domain.

Dark green = Moderate combined increasing class (N=77) with a moderate level and increasing trajectory for the inattention domain and a moderate level and stable trajectory for the hyperactivity-impulsivity domain.

Light green = Moderate combined decreasing class (N=128) with moderate levels and decreasing trajectories for both symptom domains.

Orange = Stable mild class (N=137) with mild levels and rather stable trajectories for both symptom domains.

Grey = Stable low class (N=482) with low levels and stable trajectories for both symptom domains.

Online Resource 7. Functional impairment levels over time.

The functional impairment course in all classes is plotted in Figure 3. Parent-reported functional impairment class differences were apparent at all ages. Functional impairment decreased in the severe combined stable, severe inattentive stable, and the two decreasing classes. In the moderate combined increasing class, functional impairment fluctuated, with a moderate peak in early adolescence but otherwise remaining at the same level. In the severe combined decreasing and moderate combined decreasing classes, functional impairment decreased to normative functioning levels during young adulthood. Despite improvements, the severe combined stable, severe inattentive stable, and moderate combined increasing classes continued to show some functional impairment. Self-reported functional impairment was only mildly apparent at all ages.

Figure S4. Functional impairment trajectories of parent and self-rated impact scores in participants of the longitudinal NeuroIMAGE study.

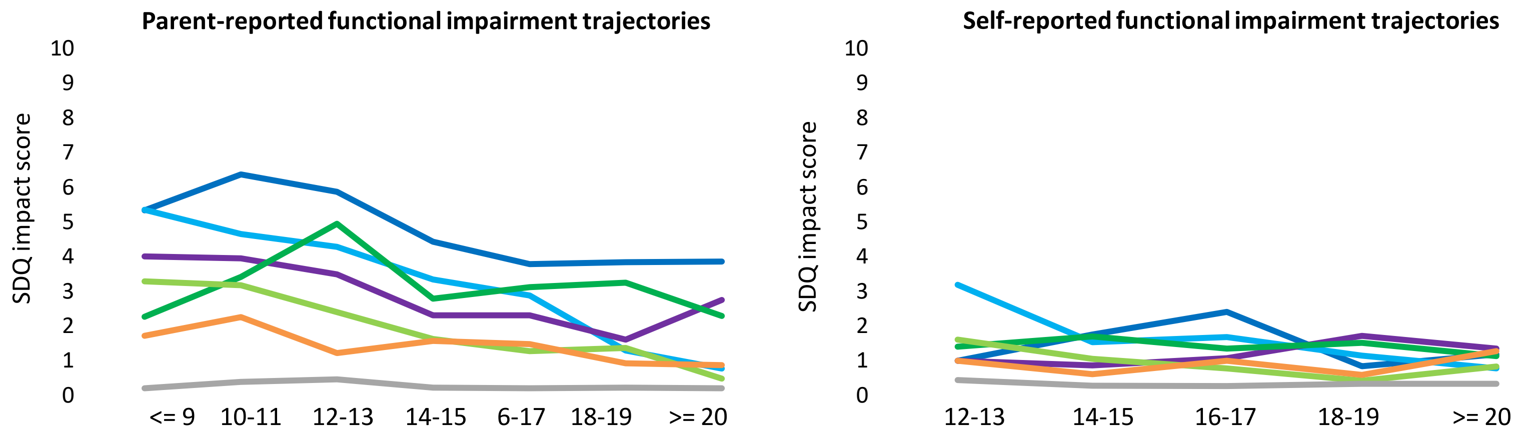

Notes: Maximum possible SDQ impact score = 10.

Dark blue = Severe combined stable class (N=48) with high levels and rather stable trajectories for both symptom domains.

Light blue = Severe combined decreasing class (N=145) with high levels and decreasing trajectories for both symptom domains.

Purple = Severe inattentive stable class (N=47) with a high level and stable trajectory for the inattention domain and a moderate level and decreasing trajectory for the hyperactivity-impulsivity domain.

Dark green = Moderate combined increasing class (N=77) with a moderate level and increasing trajectory for the inattention domain and a moderate level and stable trajectory for the hyperactivity-impulsivity domain.

Light green = Moderate combined decreasing class (N=128) with moderate levels and decreasing trajectories for both symptom domains.

Orange = Stable mild class (N=137) with mild levels and rather stable trajectories for both symptom domains.

Grey = Stable low class (N=482) with low levels and stable trajectories for both symptom domains.

Online Resource 8. Scatter plots for visual inspection of qualitative differences among the seven identified dimensional symptom trajectories of parent-rated inattention and hyperactivity-impulsivity scores in participants of the longitudinal NeuroIMAGE study.

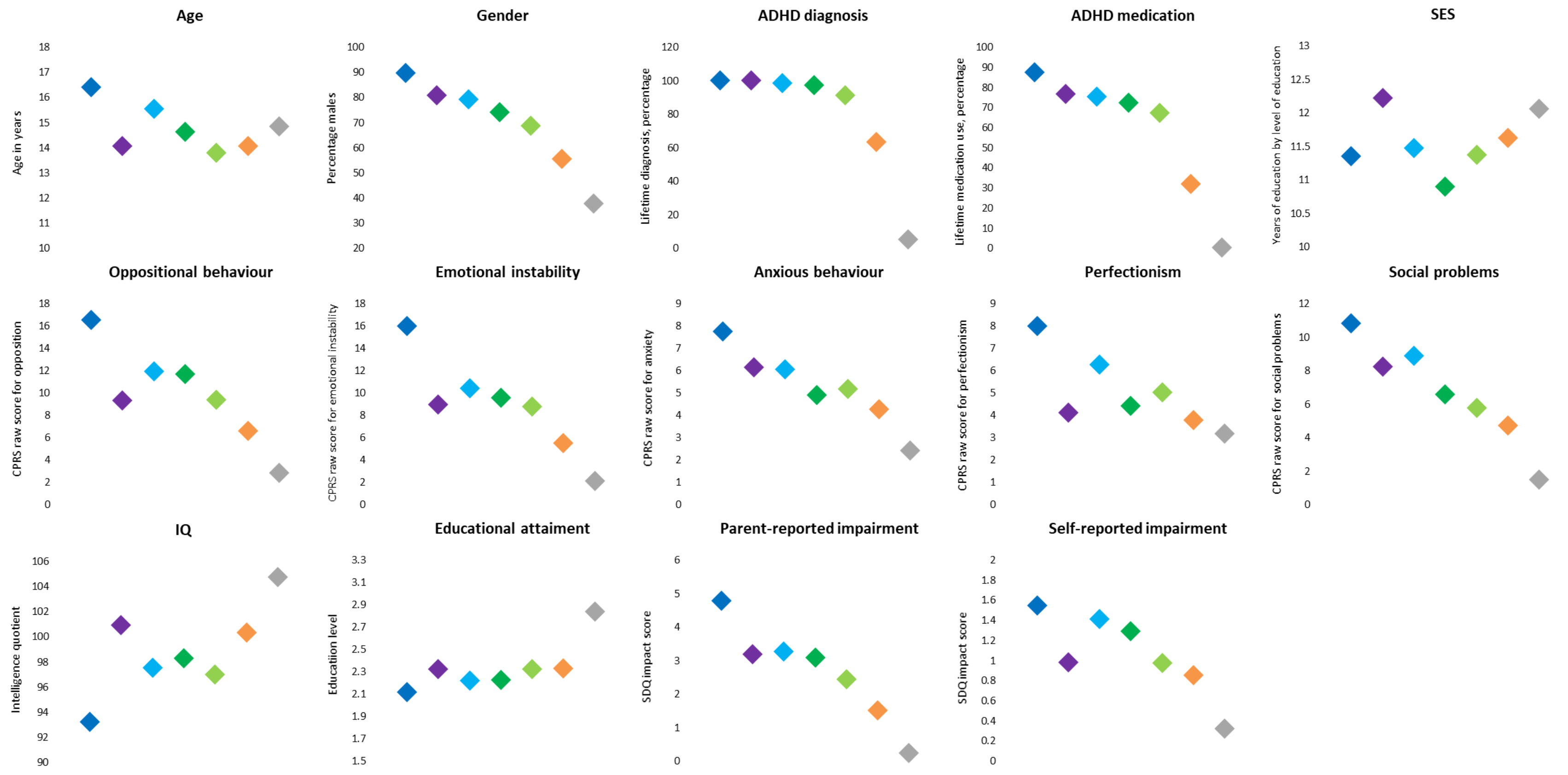

Notes: IQ = Intelligence quotient; SES = Socio-economic status.

The educational attainment score ranges from 0 ''no formal education'' to 4 ''scientific education''.

Maximum possible SES = 17; Maximum possible rescaled score for each CPRS subdomain = 30; Maximum possible SDQ impact score = 10.
